# Modeling and simulation of carbon-nanocomposite-based gas sensors

**DOI:** 10.3762/bjnano.16.9

**Published:** 2025-01-30

**Authors:** Roopa Hegde, Punya Prabha V, Shipra Upadhyay, Krishna S B

**Affiliations:** 1 Electronics and Communication Engineering, Ramaiah Institute of Technology, MSRIT Post, M S Ramaiah Nagar, MSR Nagar, Bengaluru, Karnataka 560054, India

**Keywords:** CO gas, COMSOL Multiphysics, gas sensor, surface coverage, SWCNT/PEDOT:PSS

## Abstract

This paper reports simulation of a carbon monoxide gas sensor using COMSOL Multiphysics whose active sensing material used is a carbon nanocomposite (i.e., 0.1 wt % of single-walled carbon nanotubes along with PEDOT:PSS (poly(3,4-ethylenedioxythiophene):poly(styrenesulfonate)) in an equal volume ratio of 1:1. Given the high cost associated with the development of these sensors, it becomes imperative to establish a mathematical model for economically predicting their behavior. The simulation using COMSOL Multiphysics is performed to obtain the surface coverage of the sensor by introducing carbon monoxide gas through a Gaussian pulse feed inlet at concentrations ranging from 1 to 7 ppm. The surface coverage over the range of 14% to 32.94% for the given range of concentrations is achieved giving the information of the amount of gas molecules adsorbed onto the surface of the sensing material at a given time. The surface coverage of the sensor is enhanced by using the nanocomposite materials which in turn enhances the sensitivity of the gas sensors.

## Introduction

The field of nanotechnology has brought significant advancements in various scientific and engineering disciplines, leading to the development of materials with unprecedented properties. Among these materials, carbon nanocomposites have gathered significant attention because of their exceptional electrical, mechanical, and thermal characteristics. These nanocomposites typically consist of carbon-based nanomaterials, such as carbon nanotubes (CNTs), graphene, and carbon black, embedded within a polymer matrix [1]. The distinctive properties of carbon nanocomposites have positioned them as promising candidates for various applications, particularly in the development of advanced sensors.

The small amounts of carbon monoxide (CO), an odorless, colorless, and highly toxic gas pose a serious health threat to humans. Carbon monoxide poisoning can result from incomplete burning of materials that contain carbon and produce CO. Enclosed spaces or poorly ventilated areas may accumulate CO. Symptoms of CO poisoning range from mild headaches and light headedness to sudden death or permanent brain damage. In houses as well as workplaces, it is important to ensure the constant monitoring of enclosed areas for carbon monoxide presence because of the severe risks to human health.

Traditional CO sensors, although effective, often face limitations such as poor sensitivity, slow response times, and high-power consumption. These limitations restrict their widespread use and make the development of more efficient, sensitive, and cost-effective CO gas-sensing solutions necessary. The unique properties of carbon nanocomposites, like high surface area, excellent electrical conductivity, and chemical stability, make them ideal candidates for the development of high-performance CO gas sensors. Carbon-nanocomposite gas sensors find their application in detecting pollutants such as nitrogen dioxide (NO_2_), sulfur dioxide (SO_2_), and volatile organic compounds (VOCs) in the atmosphere and can be used for industrial safety, security, and defense [2].

Gas sensors consisting of metal oxide possess several disadvantages, such as high-power consumption, sensor stability, and low sensitivity. To reduce the cost of sensors, mathematical models to predict the behavior of these sensors have been developed using numerical analysis software [1]. The polymer PEDOT: PSS aligns the surface of the CNT film by filling most of the gaps in the CNT network. The effective electrical conductivity at tube-to-tube junctions within the CNT network is increased as a result of this conductive ability of the polymer which helps CNT tubes make electrical connections [3]. Carbon nanotube sensors can be applied in various areas including environmental monitoring, biological sensors, and national security [4]. The property of the nanocomposite composed of PEDOT:PSS/poly(*p*-anisidine) (PPA) to detect CO was investigated at room temperature. The gas-sensing characteristics of the developed sensors such as sensitivity, response, and recovery time were evaluated at room temperature for different CO concentrations [5]. Many research works on PEDOT:PSS, its composites, and their application in electrochemical sensors have been discussed [6–8]. There are research contributions on pristine graphene and ammonia gas sensors for detection of very low ammonia content [9]. The thin-film sensor fabricated on polyethylenimine- (PEI) functionalized SWCNTs showed high sensitivity towards strong electron-withdrawing molecules for NO_2_ gas detection [10]. To simulate gas adsorption in COMSOL Multiphysics for a gas sensor, several adsorption models can be employed depending on the physical and chemical behavior of the sensor. Commonly used models include the Langmuir adsorption model, Freundlich adsorption model, Temkin adsorption model, and Brunauer–Emmett–Teller (BET) model. The Langmuir adsorption isotherm equation is typically derived through a kinetic approach and relies on certain assumptions [11]. Accurate adsorption processes rely on effective isotherm modeling and interpretation. Although linear regression is widely used to assess fit quality, nonlinear regression is increasingly favored for better matching between predicted and experimental data, underscoring the importance of evaluating both methods in adsorption systems [12]. The Langmuir adsorption model was initially formulated to characterize gas adsorption on solid-phase adsorbents such as carbon-based adsorbents [13]. The fundamental assumptions of the Langmuir isotherm include: (1) adsorption occurs as a monolayer; (2) adsorption sites are uniformly distributed; (3) the adsorption energy remains constant; and (4) interactions between adsorbate molecules are insignificant [14]. Parameter estimation is typically conducted using linearization methods. This study evaluates the applicability and accuracy of different linearization approaches for the Langmuir isotherm [15]. There are some studies related to examining the suitability and limitations of the Langmuir adsorption isotherm for major unconventional gas resources [16]. The Langmuir isotherm is suitable for modeling monolayer adsorption on uniform sites. Fitting experimental adsorption data to the appropriate isotherm model is crucial for understanding the adsorption mechanism in a system and predicting its behavior [17].

## Methods

### Gas sensor analysis with COMSOL Multiphysics

**Figure 1:**
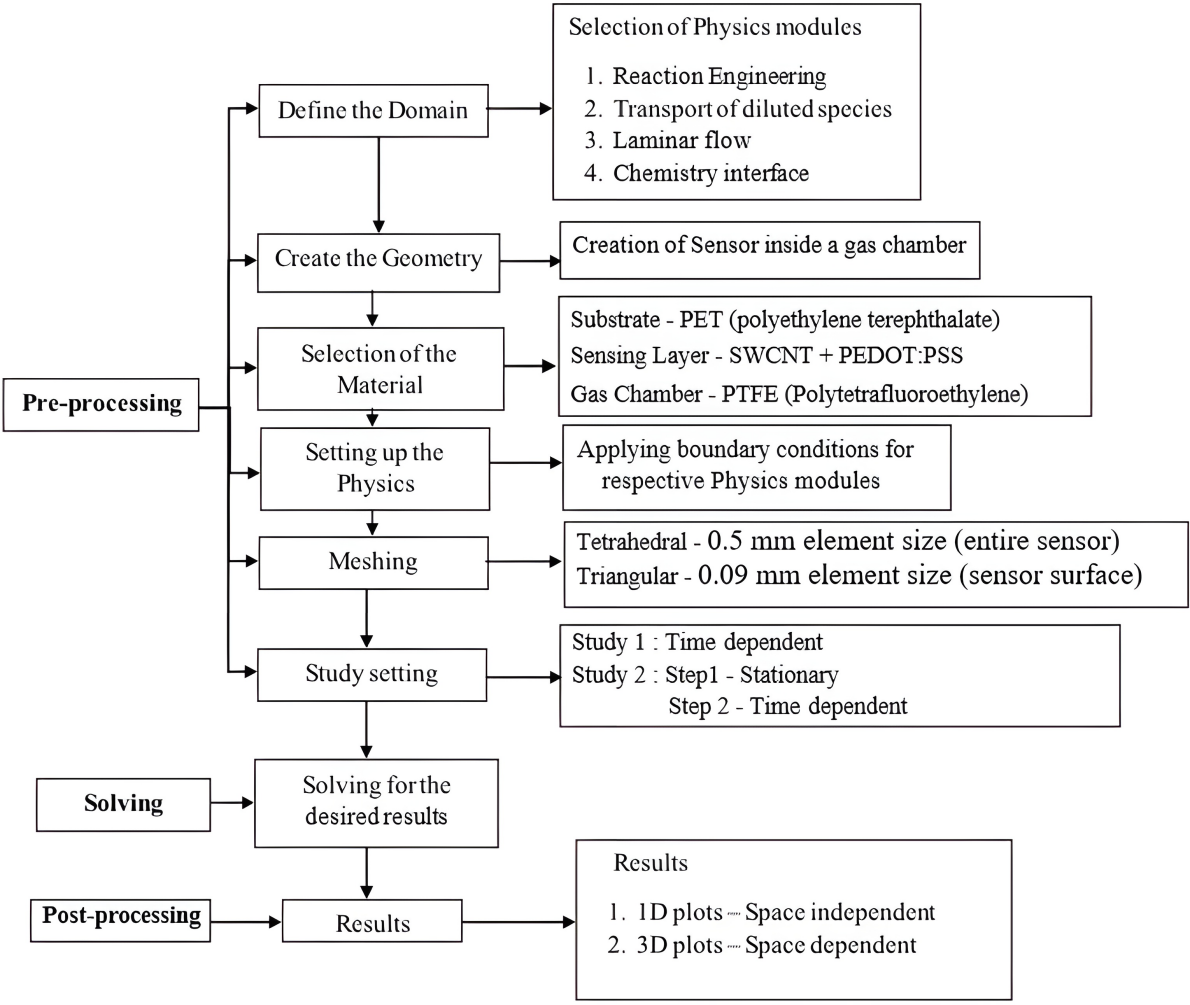
Steps followed in implementing the sensor.

To implement the methodology from the flow chart in Figure 1, we begin with pre-processing. During this step, we define the domain with physics modules (reaction engineering, transport of diluted species, laminar flow, and chemistry interface), create the geometry of the sensor within a gas chamber, and select the materials. We then set up the physics with boundary conditions, mesh the geometry using tetrahedral and triangular elements, and define study settings for time-dependent and stationary analyses. In the solving phase, simulations are executed to generate results, followed by post-processing to analyse and present the outcomes in 1D and 3D plots.

The sensor layer where the adsorption of the gas occurs and thus participate in detecting the CO gas is made up of a carbon nanocomposite material, and the material properties considered in this project are as shown in Table 1.

**Table 1:** Properties of the carbon nanocomposite used in this work.

Density	1004 kg/m^3^

Young's modulus	2.77 × 10^9^ Pa
electrical conductivity	11.97 S/m
relative permittivity	2.3
diffusion coefficient	2 × 10^−5^ m^2^/s
Poisson's ratio	0.5
thermal conductivity	0.3 W/(m·K)
coefficient of thermal expansion	7 × 10^−5^ 1/K
dynamic viscosity	1.8 × 10^−6^ Pa·s

### Gas chamber modeling

The sensor is placed within an enclosed chamber which consist of two outlets and a single inlet at the top of the chamber. This chamber is specifically designed to facilitate gas flow and continuous stirring, ensuring the elimination of concentration gradients and the maintenance of a homogeneous gas mixture inside the chamber. The target gas CO is introduced into the chamber in a Gaussian pulse form. At the start of the experiment (*t* = 0 s), the sensor surface is assumed to be fully covered by ambient air molecules.

**Figure 2:**
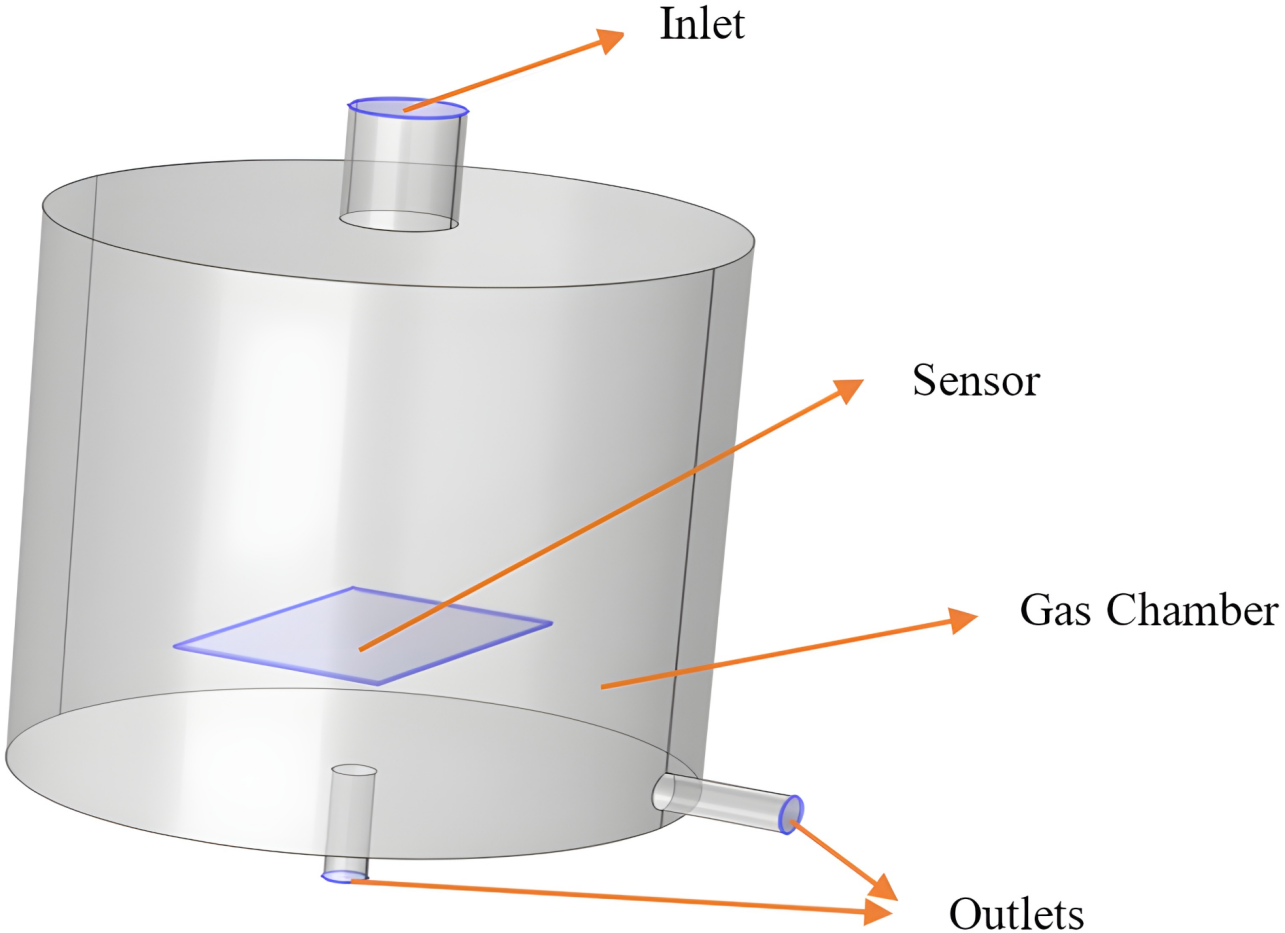
The gas chamber with a sensor.

**Figure 3:**
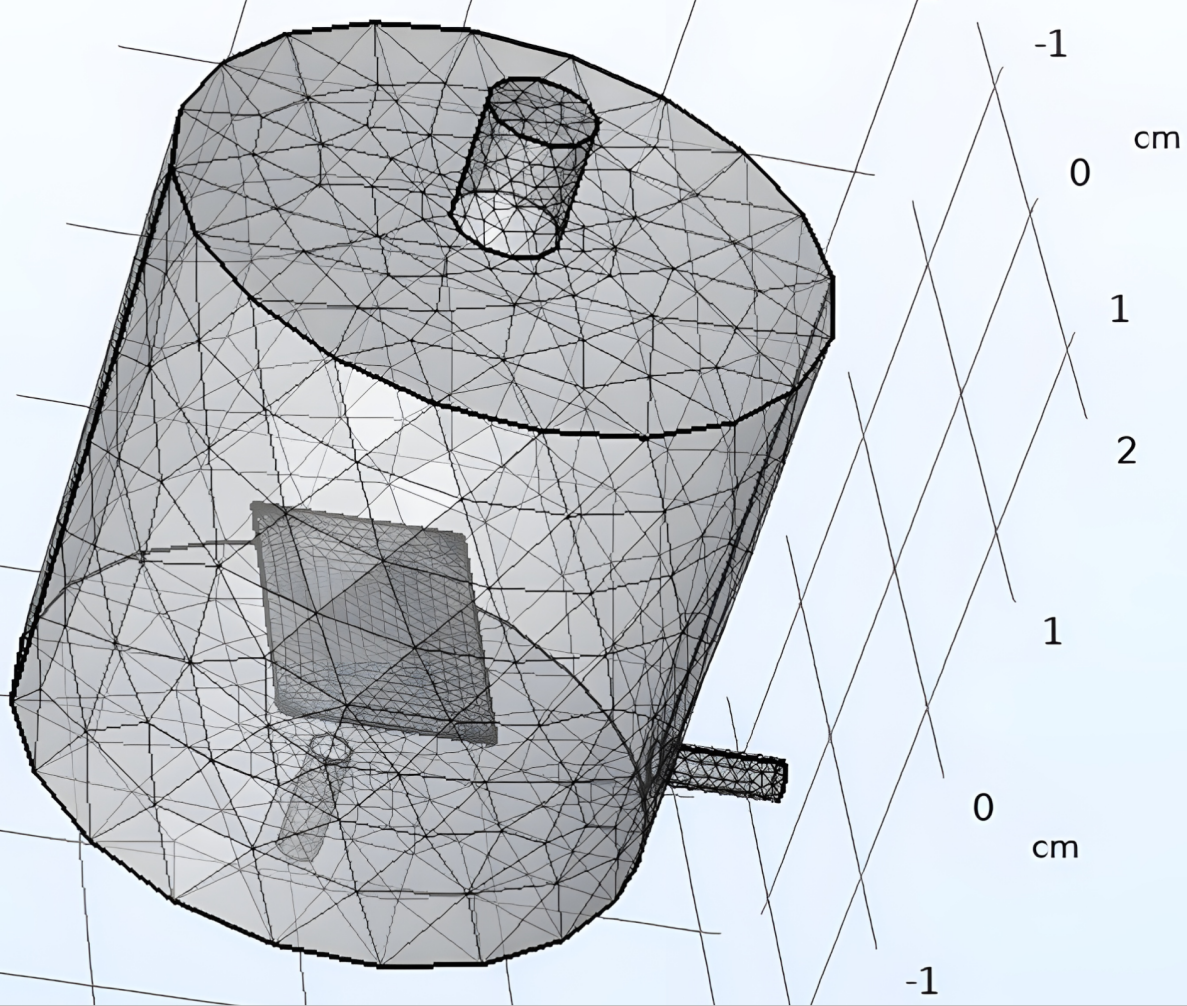
Meshing of the gas chamber with a sensor.

The sensor is modeled as a block (10 mm × 15 mm × 0.125 mm) which is then placed inside a chamber with a radius of 1.5 cm and a height of 3 cm. The inlet with diameter of 0.5 cm and two outlets with a diameter of 0.2 cm each having a length of 0.6 cm are built as shown in Figure 2. Meshing is a crucial step in the simulation process, dividing a large domain into smaller parts to apply boundary conditions. For this model, tetrahedral elements with an element size of 0.5 mm were used as the primary mesh element throughout the body. Additionally, a second mesh node was added for the sensor layer to create a refined mesh of triangular elements with an element size of 0.09 mm. The meshing settings in the COMSOL software are illustrated in Figure 3.

Table 2 lists the parameters used in the simulation to examine how surface coverage varies with changes in CO concentration. Surface coverage in the nanocomposite is governed by the adsorption of gas molecules. When CO molecules come in contact, they react with these adsorbed oxygen ions, which releases electrons into the material and alters its electrical properties.

**Table 2:** Parameters used during simulation.

Parameter	Value	Description

Arsurf	1.5625 × 10^−4^ m^2^	surface reaction area
vfp	2.1208 × 10^−5^ m^3^/s	volumetric feed to the inlet
CAmax_inlet	0.0357 mol/m^3^	maximum CO concentration in the inlet
CBmax_inlet	0.03125 mol/m^3^	maximum O_2_ concentration
CS0surf	9.9 × 10^−6^ mol/m^2^	initial S surface concentration
G0	2.3 × 10^−5^ mol/m^2^	initial site density of reactive surface
U_column	1 × 10^−4^ m/s	velocity of gas
MA	0.02801 kg/mol	molar mass of CO
MB	0.031998 kg/mol	molar mass of O_2_
MS	0.318 kg/mol	molar mass of S

## Results and Discussion

### Space independent analysis at 1 ppm of CO gas

**Figure 4:**
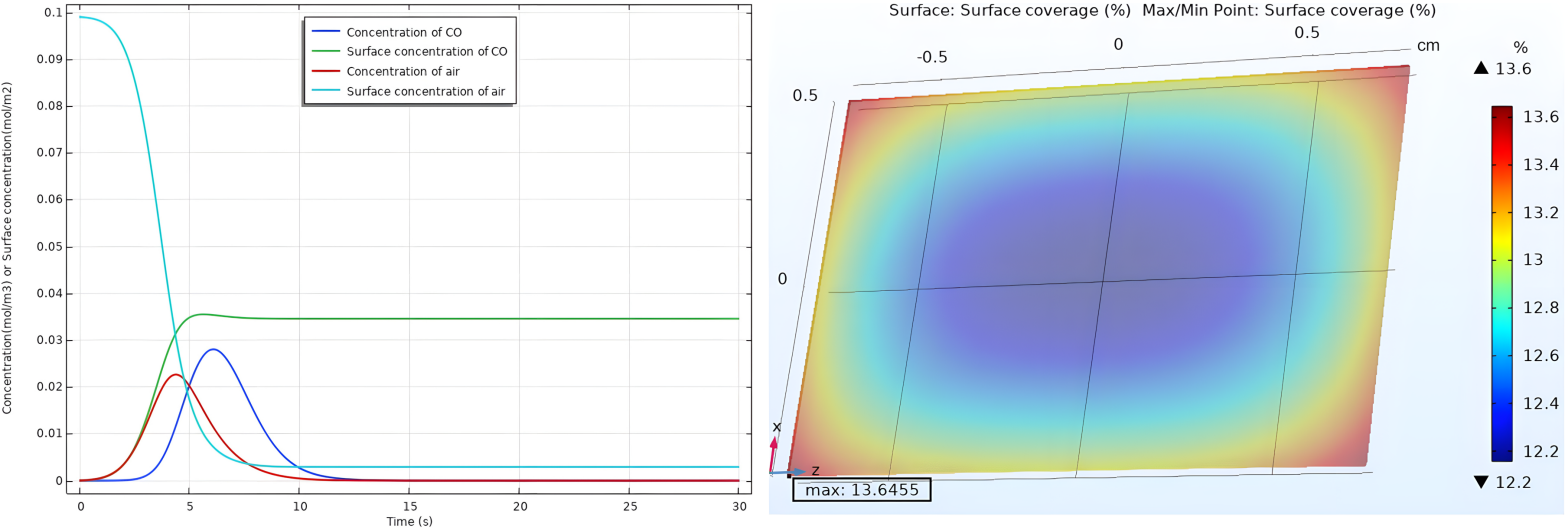
Surface concentration and surface coverage (θ) at 1 ppm of CO gas concentration.

Figure 4 depicts the temporal changes in reactant concentrations over time. Initially, at *t* = 0 s, no gases are injected into the chamber, resulting in zero concentration for all gases. However, air is already adsorbed on the sensor surface, leading to its maximum surface concentration. At *t* = 6 s, injection occurs via a Gaussian concentration pulse feed inlet for 1 ppm CO gas concentration, characterized by a bell-shaped curve. This indicates that during the injection process the concentration gradually rises to a peak before returning to the baseline within a period of 9 seconds. As the CO gas adsorbs onto available sites in the sensor layer, air molecules begin to desorb from the surface. Consequently, the surface concentration of air decreases while the surface concentration of CO gas increases until it reaches a steady state. Active sites on the surface of the sensor are considered by defining the reaction area and the surface reaction module.

The color scale moves from blue (lower values) to red (higher values), with shades of green, yellow, and orange in between to represent intermediate coverage levels. The lowest coverage (12.2%) is represented by the deep blue regions, whereas the highest coverage (13.6%) is represented by the bright red regions. The gas entering the chamber experiences a pressure gradient from the inlet point to areas farther away. This pressure gradient causes the gas to radially expand, spreading out toward the cylinder walls as it moves downward.

### Space dependent analysis at different concentrations

**Figure 5:**
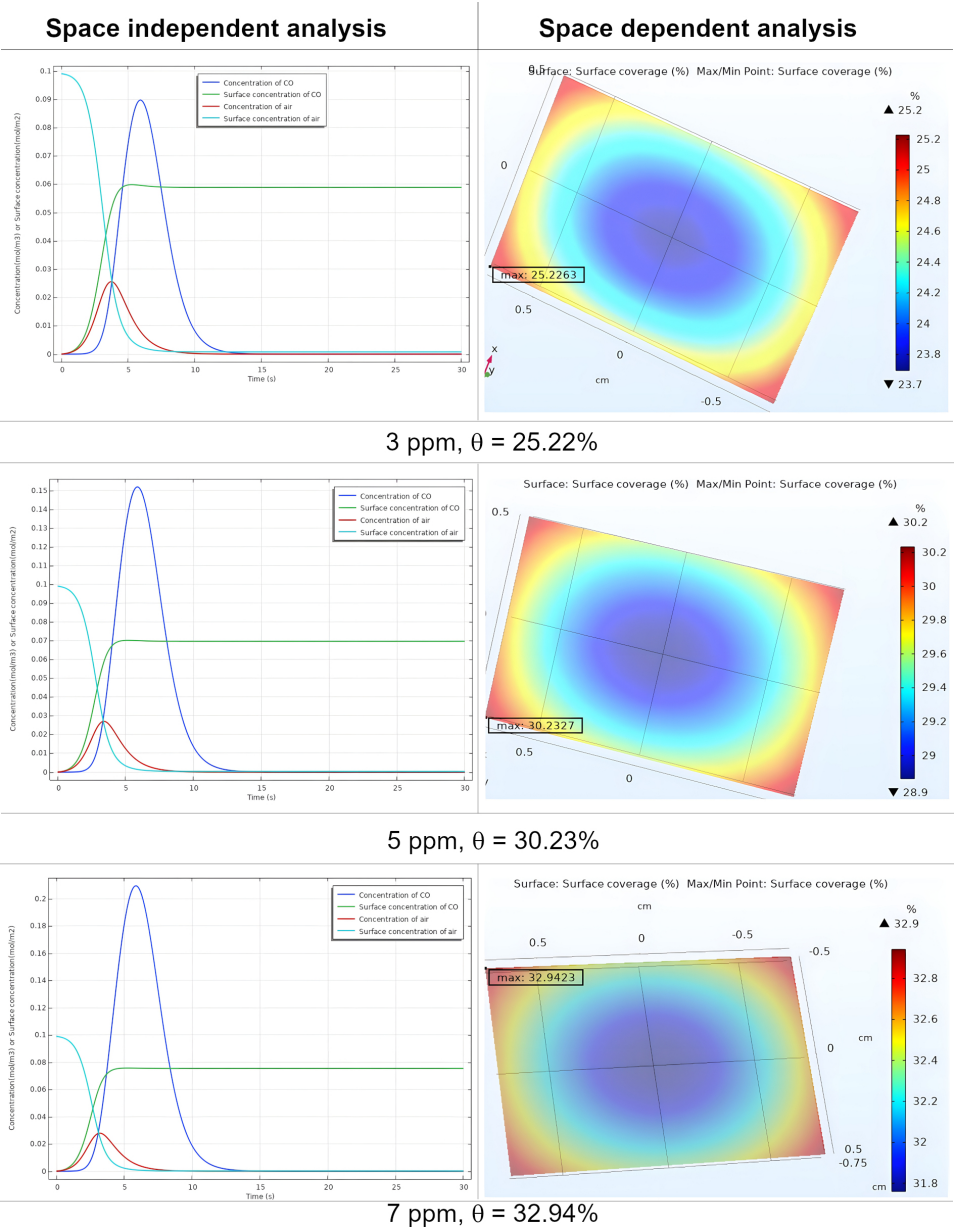
Surface concentration and surface coverage (θ) at concentrations of 3, 5, and 7 ppm of CO.

Figure 5 depicts 3D plots illustrating the maximum surface coverage of CO on the nanocomposite surface at various concentrations of the target gas. At 1 ppm, the adsorption is notably uniform, achieving a coverage of 14% due to ample active site availability. However, at higher concentrations such as 3 (25.22% of coverage), 5 (30.23% coverage), and 7 ppm (32.94% of coverage), the uniformity of adsorption decreases. This is attributed to the limited availability of active sites on the surface. The plots demonstrate that an increase in gas concentration within the test chamber corresponds to an increase in the number of adsorbed molecules on the surface, thereby enhancing surface coverage.

**Figure 6:**
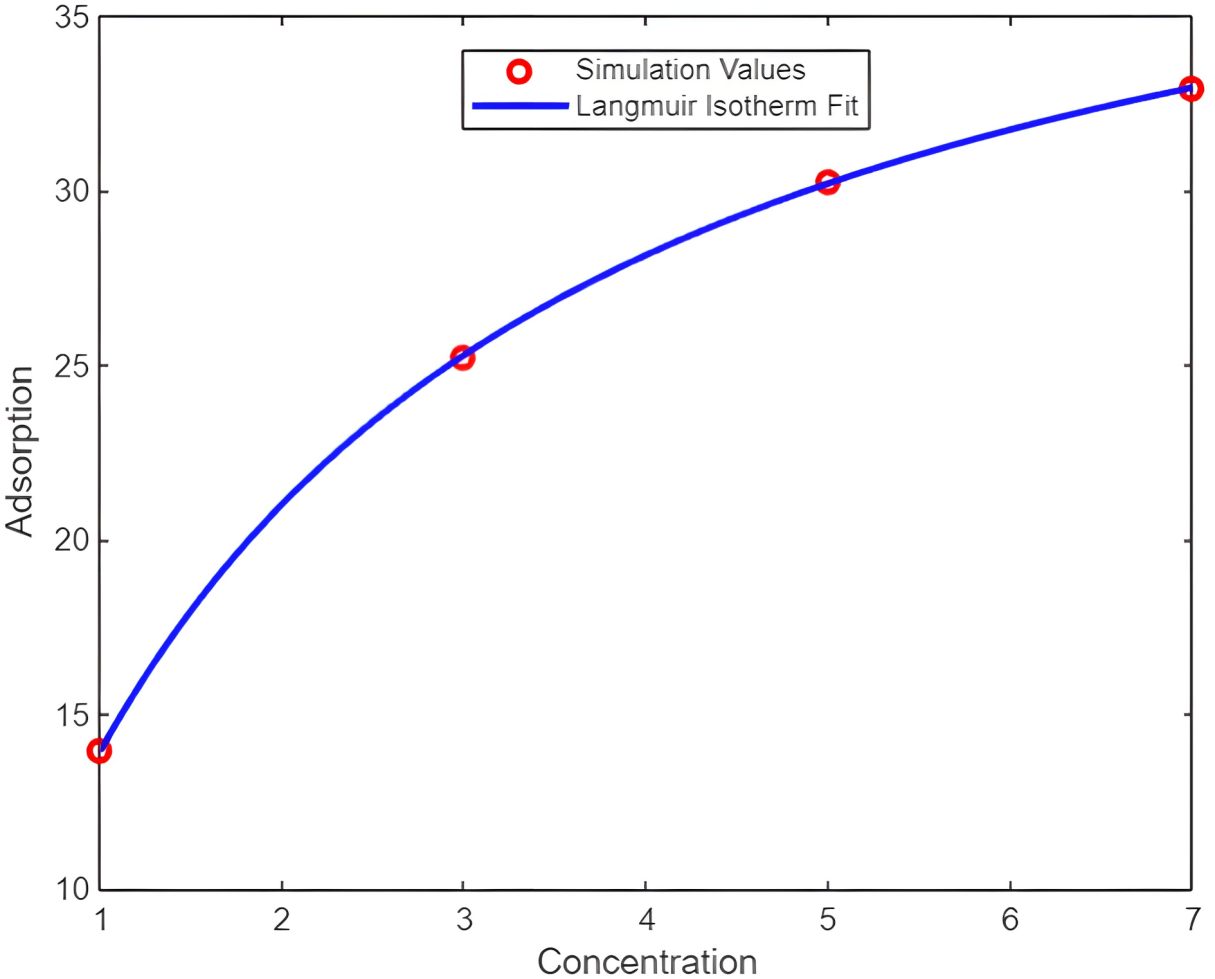
Langmuir adsorption model.


The Langmuir adsorption model is used which is suitable when one assumes a monolayer of adsorbed gas molecules on a homogeneous surface. It is often used in gas sensors where only one layer of gas molecules is adsorbed and where there are no interactions between adsorbed molecules. Fitting of obtained result values into the Langmuir adsorption model is shown in Figure 6, which is defined using the Equation 1.

[1] $$\theta =\frac{KC_{\text{e}}}{1+KC_{\text{e}}},$$

where *K* is the affinity constant (L/mol), θ is the surface coverage, and *C*_e_ is the solution concentration at equilibrium (mol/L).

## Conclusion

The study effectively demonstrates the simulation of a CO gas sensor using COMSOL Multiphysics to assess the surface coverage of the sensor, a key factor regarding sensitivity. The active sensing layer, a carbon nanocomposite of SWCNTs and PEDOT:PSS, is shown to achieve surface coverage between 14% and 32.94% across CO concentrations from 1 to 7 ppm. This range indicates the ability of the sensor to adsorb gas molecules at varying levels of CO exposure. Comparisons with the Langmuir isotherm model indicated good alignment, suggesting potential for accurate predictive modeling. The simulation provides an economical approach to predict the behavior of the sensor, reducing the costs associated with sensor development. Overall, these findings lay a robust groundwork for advancing CO gas sensor technology, promising more effective solutions for environmental and industrial safety applications.

## Data Availability

Data generated and analyzed during this study is available from the corresponding author upon reasonable request.
